# An optimal painless treatment for early hemorrhoids; our experience in Government Medical College and Hospital

**Published:** 2013-09-25

**Authors:** R Singal, S Gupta, AK Dalal, U Dalal, AK Attri

**Affiliations:** * Department of Surgery, Maharishi Markandeshwer Institute of Medical Sciences and Research, Mullana (Distt-Ambala) Haryana, India; ** Department of Radiodiagnosis and Imaging, Maharishi Markandeshwer Institute of Medical Sciences and Research, Mullana (Distt-Ambala) Haryana, India; *** Department of Surgery, Government Medical College and Hospital, Chandigarh, Punjab, India; **** Government Medical College & Hospital, Chandigarh, Punjab, India

**Keywords:** Anal bleeding, hemorrhoid, Non-surgical, photocoagulation, ambulatory

## Abstract

Objective - To evaluate the efficacy of Infrared Coagulation Therapy (IRC) for hemorrhoids. IRC is a painless, safe and successful procedure.

Place and duration of study - Department of Surgery, Government Medical College and Hospital, Sector-32, Chandigarh, India, from August 2006 to October 2008. The choice of procedure depends on the patient's symptoms, the extent of the hemorrhoidal disease, and the experience of the surgeon along with the availability of the techniques/instruments.

Materials and methods - This is a prospective study done from August 2006 to October 2008. Total number of 155 patients was included in the study. Infrared Coagulation Therapy (IRC) was performed through a special designed proctoscope. Patients excluded were with coagulopathy disorders, fissure in ano, and anal ulcers.

Results - It is an outpatient Department (OPD), non-surgical, ambulatory, painless and bloodless procedure, without any hospital stay. Early recovery and minimal recurrence of hemorrhoids were noted without any morbidity or mortality. We have studied 155 patients, treated with IRC on OPD basis. Surgery was required in few patients in whom IRC failed or was contraindicated.

Out of the total 155 patients, 127 came for follow up. After the 1st sitting of IRC therapy: out of 127; 43 patients got a total relief, mass shrinkage was of > 75% in 57 cases and < 50% in 14 cases. Twenty-eight cases did not come for follow-up. In the 2nd sitting, out of 84/127; 58 patients got a total relief, >75% relief in 15 cases and >50 % relief in 11 patients. In the 3rd sitting out of 26/84 cases: 13 cases got a total relief and 13 cases refused to take the third sitting; however, in 7 cases the hemorrhoidal mass shrank up to 50% after the two sittings. These 14 were operated as there was no relief from bleeding after giving two sittings of IRC. Our opinion is that, in the above 14 cases, the patient might have not followed the instructions properly for dietary habits.

Conclusion - IRC is a safe, simple and effective procedure for early hemorrhoids without any complications. IRC is nowadays the world’s leading office treatment for hemorrhoids. IRC is a better option than the surgical treatment as it is easy, well tolerated, and remarkably complication-free. In our study, we have not used any course of antibiotics. In the management of early hemorrhoids, IRC should be considered as a simple trouble-free and painless option.

## Introduction

Hemorrhoids arise from the engorgement of the submucosal anal cushion, which is made up of an interlacing arteria-venous hemorrhoidal plexus, supported by connective tissue and minute muscle fibers [**1**]. These are one of the frequently occurring diseases that should be treated as early as possible. The most commonly used treatment options are injection sclerotherapy, rubber band ligation, surgery, etc., but non-surgical modalities such as Infrared coagulation (IRC/Photocoagulation) reduces the post-operative pain, so is frequently used nowadays [**2**]. IRC is one of the latest outpatient department (OPD), safe, easy, short duration procedure with early recovery. The first surgical treatment was described in the Hippocratic treatise of 460 BC and suggested to transfix them. The IRC technique for thermal ablation of hemorrhoids was first described by Neiger [**3,4**]. It is an easy, non-invasive, painless, safe method of treatment, giving results that are comparable to the other treatment options with the avoidance of surgery. In our study, patients treated with IRC came out with good results. 

### Aims and objectives

 The aim of this study was to assess complications and long-term outcome of IRC to make patients symptom-free and decide whether IRC can be used as an alternative to the other treatment options. It can also be used in complicated cases where surgery remains contraindicated. It is a non-invasive procedure for Ist and IInd grade symptomatic hemorrhoids and few cases of IIIrd and IVth grade in which surgery is contraindicated.

## Material and methods

The study was carried out prospectively from August 2006 to October 2008. The total number of patients selected was 155, diagnosed as Ist and IInd grade hemorrhoids based on history and clinical examination, including proctoscopy. 61 patients had Ist grade and the remaining 94 had IInd grade hemorrhoids. Out of the total 155 patients, 83 had already received conservative treatment but failed to respond. All patients were given the IRC treatment. The age criteria included was 14 to 85 years.

 Procedure

**Fig. 1:**
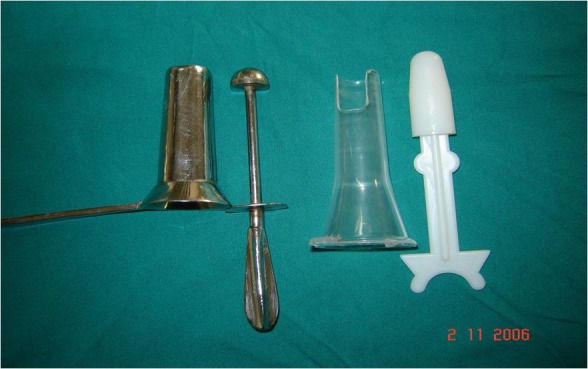
Proctoscope used for hemorrhoids

 IRC was performed through a special designed proctoscope, which has a simple vent on one side, through which single hemorrhoidal mass protrudes out and compresses the other hemorrhoidal masses (**Fig. 1**). An informed consent was taken from all the patients. The patient was placed in the left lateral decubitus and 3 to 4 pulses of the IRC were applied at the base of the hemorrhoids for 1.5 seconds. Local 5% xylocaine jelly was applied over the anus and proctoscope, which reduces the sensitivity of the area and facilitates the passage of the anoscope.

 The IRC apparatus consists of a power supply unit and an applicator/probe, which facilitates the treatment through an anoscope (Courtesy of Redfield Corporation; Montvate: New Jersey and infrared coagulator, LUMBATEC: Germany). IRC is closely related to coagulation by laser beam and developed as a spin-off of laser technology. 

**Fig. 2:**
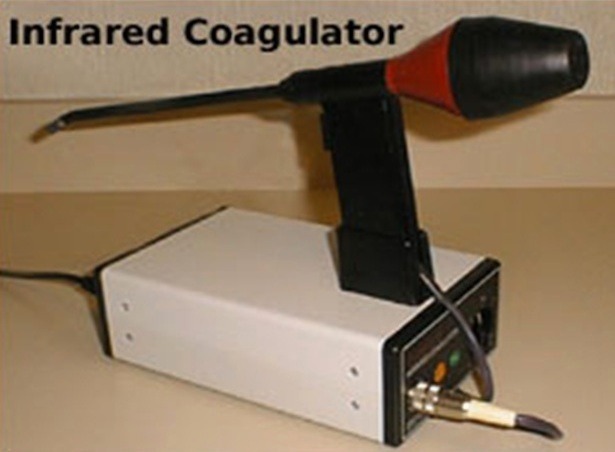
Device, which helps in the coagulation therapy (Courtesy of Redfield Corporation Montvale. New Jersey)

 The technology involves the infrared radiation generated by a tungsten halogen lamp and mounted on an instrument that resembles a gun with a trigger (**Fig. 2**). It is a non-operative procedure, which uses infrared energy to create thrombosis, submucosal fibrosis, leading to mucosal fixation, which causes scarring of the hemorrhoidal tissue and leads to the reduction of the mass. Injection sclerotherapy is another non-surgical modality performed through a proctoscope in the left decubitus position. Injection was given submucosally up to 5 ml of 5% phenol in arachis oil, into the base of each hemorrhoid above the dentate line. However, it has its own disadvantages – pain and bladder complications and infection. 

 However, in IRC, the hemorrhoidal veins shrink and cause little pain and in most of the cases, it is entirely painless. In our study, 14 months of follow up was done. We have documented the outcome, the patient’s response (complete relief of symptoms versus no change in symptoms), requirement of re-treatment and complications like bleeding and pain. In IRC therapy one, two or all three of the internal hemorrhoids, masses can be treated in one session and can be repeated two to four times with therapeutic window of 21 days without encountering any complications. 

 Exclusion criteria 

 Patients excluded were with coagulopathy disorders, fissure in ano, and anal ulcers. Those patients diagnosed as grade III and IV hemorrhoids were also excluded, except for those who were not fit for surgery. Patients were followed up for 14 months.

## Results

IRC can be considered a safe, painless and simple OPD procedure for the treatment of hemorrhoidal disease with excellent long-term results. It is a bloodless, non-surgical procedure with early recovery and minimal recurrence. Surgery was required in some patients in whom Infrared Coagulation Therapy (IRC) failed or was contraindicated. 

 Out of the total 155 patients, 127 came for follow up. After the 1st sitting of IRC therapy: out of 127; 43 patients got a total relief, mass shrinkage was > 75% in 57 cases and < 50% in 14. In the 2nd sitting, out of 84/127; 58 patients got a total relief, >75% relief in 15 cases and >50 % relief in 11 patients. In the 3rd sitting, out of 26 cases: 13 cases got a total relief and 13 cases refused to take the third sitting; however, in 7 cases, the hemorrhoidal mass shrank up to 50% after the two sittings. These 14 were operated as there was no relief from bleeding after giving two sittings of IRC (**Table 1**). Our opinion is that, in the above 14 cases, the patient might have not followed the proper instructions for dietary habits.

**Table 1:** No relief from bleeding after giving two sittings of IRC

IRC	Follow-up	Results	Operated
Ist sitting	Total patients – 155 follow-up- 127	- 43 patients got total relief; -Mass shrinkage - >75% in 57 cases and < 50% in 14 cases - 13 cases had minor relive(bleeding was present)	
2nd sitting	84/127	<- 58 cases got total relief >90% -mass shrinkage - > 75 % relief in 15 patients and <50 % in 11 case (total-26)	
3rd sitting	26/84	-total relief in 13 patients - 13 cases refused to take 3rd course	13 patients refused to get 3rd course of therapy so operated
total patients	155	127 came for follow-up	

## Discussion

 The history of ligation of the pile mass is quite old. Hemorrhoids are nowadays a common problem worldwide. Data from the National Center for Health Statistics suggest that approximately 10 million people in the United States suffer from hemorrhoids. 

 The word hemorrhoids [haima =Blood: rhoos = flowing], derives from the Greek adjective, hai. Hemorrhoids are the clinical manifestation of the downward disruption of normal functional structures known as the anal cushions. In 1975, Thomson published his master’s thesis based on anatomic and radiologic studies and introduced the term vascular cushions [5,6]. 

 There are different types of treatment options for hemorrhoids, both operative and non-operative. IRC (also called photocoagulation therapy) is a non-surgical, medical procedure commonly used for Ist and IInd grade hemorrhoids and also in grade III cases which remain unfit for surgery. Two meta-analyses were published. In 1992, Johanson JF studied that IRC is the best non-operative therapy and, in 1995, Mac Rae declared that Rubber band ligation (RBL) should be the optimal treatment for 1st, 2nd, and 3rd grade hemorrhoids [**6**]. IRC causes less pain than Rubber band ligation but their short-term clinical results are similar [**6**]. Hardy A et al [**3**] studied that IRC has good results than injection sclerotherapy. Radiofrequency coagulation can be used in pregnant patients where surgery is contraindicated as we did IRC in two cases without any complications. IRC is a safe and swift procedure for internal hemorrhoids. 

 The infrared radiation (IR) is defined as an electromagnetic radiation that has a wavelength longer than the visible light, but shorter than that of microwaves. Do not let the term “radiation" scare you, infrared radiation is not the same as the radiation that nuclear plants put out. In 1981, Leicester [**4**], found the IRC to be a simple, rapid, non-invasive method of treatment for hemorrhoids that is safer and less painful than the conventional techniques. IRC also enjoys the added advantage of not causing high frequency electromagnetic interference, which could affect implanted devices such as the pacemaker.

 Side effects to the technique:

 Pain - The patient feels some intra-anal discomfort, which can persist for several days after the IRC reported incidence is 20-35%, but 9 to 70% by injection sclerosis, and with banding 5 to 85% [**7**]. In our study, there was no post-operative pain.

 Rectal bleeding - It is seen in 2-10% of cases after sclerotherapy, 1 to 15% after rubber band ligation and 5 to 25% after infrared coagulation. Ligation can result in pain, external hemorrhoidal thrombosis and internal dysuria in 1-5% of cases [**8**]. 

 The other rare complications are hemorrhage or infection, urinary complaints, etc. The heavy rectal bleeding, requiring transfusion or surgical hemostasis, usually occur during the fall of pressure ulcers between 5th and 12th days. They are mostly described after ligation (0.5% to 2% of cases) but may occur after the techniques of coagulation, monopolar and bipolar especially during heavy use (0% to 8% of cases) [**9**]. In our study, there were no complications like infection, stricture, bleeding, or any procedure related odor, in the follow up period of 14 months.

 RBL should be mainly used for internal IInd grade hemorrhoids, and sclerotherapy is indicated to decrease acute bleeding, but in the long-term, this method has the poorest results. IRC has its good results in internal Ist grade hemorrhoids because it causes less pain and complications and patients accept it better [**10**]. Patients should be told about the early signs of complications like the onset of pain, with or without fever, with urinary symptoms. Antibiotic prophylaxis with metronidazole is recommended as a prescription painkiller of class 1 or 2, but we did not give any antibiotics except for analgesics for two days [**11**]. The Chew et al combined injection sclerotherapy with RBL achieved 90 percent of success. The complication rate was of 3.1 percent with an overall recurrence rate of 16 percent. Only 7.7 percent of these patients required hemorrhoidectomy [**12**]. The complications can be decreased by using a proper technique and making office treatment for first to third grade hemorrhoids tolerable and satisfying [**2**].

 RBL is probably the most commonly used non-surgical treatment for hemorrhoidal disease. IRC is one of the most recent advances based on the use of thermocoagulation. Recent studies have demonstrated similar efficacy for both modalities. They concluded that both RBL and IRC are best methods for the internal hemorrhoids; in addition, both procedures had relatively minor complications [**13**].

 In total, for Ist and IInd grade hemorrhoids, the practitioner has a wide range of choice and can choose an expertise treatment. In prolapsed IIIrd grade hemorrhoids, only the elastic ligatures have proved effective in three quarters of the cases [**14**]. In a retrospective study, Gupta PJ described that IRC is better tolerated than band ligation for Ist and IInd grade hemorrhoids and could be an easy and effective alternative to conventional treatment, as in our study which showed similar results [**15**].

 Kaman L et al reported a patient who underwent submucosal injection sclerotherapy for hemorrhoids and presented with necrotizing fasciitis of the anorectum, perianal region and scrotum. Post-operatively, the patient developed septicemia and renal failure requiring an extended hospital stay [**16**]. Septic complications following both conservative and surgical treatment of hemorrhoids are rare but may be catastrophic. Immunological compromise poses an additional risk for many treatment modalities [**17**]. Patients should be informed about early signs of cellulitis like pelvic pain, urinary and anorectal problems. Antibiotic prophylaxis is framing the action recommended by expert consensus. IRC should be considered as a safe, simple, trouble-free option for the symptomatic hemorrhoidal cases. There was an improvement in 73% of the patients. Fifty-nine percent of the patients became asymptomatic and 14% of the patients had a partial improvement with a reduction in bleeding and prolapse. No response was seen in 15% of the cases [**18**].

 In addition, like any surgical procedure, IRC for hemorrhoids carries a slight risk of shock or infection. These last two are rare, but if they occur, they need to be treated immediately. The recovery time is usually one to two weeks after IRC. We advised analgesics for two days, and diet therapy to avoid constipation and in addition advised to stop the exercise or physical exertion. Our study results showed a painless, non-infectious and uncomplicated procedure.

 Advantages: 

 - procedure completes in a short span of time, and the recovery time is also short

 - all the hemorrhoids can be treated in one sitting as an outpatient

 - it is beneficial for those cases that have not responded well to other treatments

 - finally it is less painful than other methods, such as band ligation

 - IRC can bring amazing results in other fields like skin, cosmetic surgery and ENT, etc.

 While the patient may not be able to exercise as much as his likes during his recovery, he has to keep up with the dietary and water intake changes. When he is able to do it, exercise is added back into his routine to get the best possible defense against hemorrhoids and a myriad of other health problems.

## Conclusion

 IRC is easy to implement, an ambulatory instrumental treatment for hemorrhoidal disease. IRC procedure is one of many final resort solutions to a problem that is estimated to affect three out of four people at some time during their life. Ist and IInd grade as well as relatively small IIIrd grade hemorrhoids can be managed non-operatively. We did not encounter any complications. IRC should be considered as a simple trouble-free, safe, non-surgical and outdoor option.
